# Diagnostic Opportunities for Optimizing Management of Multidrug-Resistant Tuberculosis (MDR-TB) in Tanzania

**DOI:** 10.24248/EAHRJ-D-16-00357

**Published:** 2018-04-01

**Authors:** Stellah G Mpagama

**Affiliations:** a Kibong'oto Infectious Disease Hospital, Kilimanjaro, Tanzania

## Abstract

**Background:**

Tanzania is one of the countries confronting a multidrug-resistant tuberculosis (MDR-TB) epidemic.

**Research:**

Research studies on drug susceptibility testing (DST) for second-line TB drugs given to Tanzanian MDR-TB patients has demonstrated mycobacterial resistance to important MDR-TB drugs, such as ethionamide, ofloxacin, amikacin, kanamycin, and pyrazinamide. Likewise, pharmacokinetic studies have shown a high frequency of patients with circulating serum drug levels below the expected ranges, especially for levofloxacin and kanamycin – key drugs in MDR-TB treatment that also affect ex-vivo plasma drug activity.

**Recommendations:**

We suggest using molecular diagnostic assays, such as the GenoType MTBDRplus test, and *inhA* and/or *katG* genotypic results to optimize MDR-TB treatment. Quantitative drug susceptibility can guide the selection of options for second-line anti-TB drugs. The TB drug assay, an alternative biomarker for therapeutic drug monitoring, can identify patients who have extensively drug-resistant TB or are exposed to suboptimal serum drug levels of, specifically, levofloxacin and kanamycin.

## BACKGROUND

Multidrug-resistant tuberculosis (MDR-TB) is a public health crisis requiring novel approaches to diagnosis and treatment.^1^ In many resource-limited settings, treatment is empirical and not based on known *Mycobacterium tuberculosis* (MTB) susceptibility patterns to the drugs that comprise the multidrug treatment regimen. The empirical MDR-TB regimen recommended by the World Health Organization (WHO) consists of pyrazinamide – which belongs to the first-line TB regimen, or group 1 TB drugs – and at least 4 other second-line TB drugs.^2^ The second-line drugs are categorized in different groups, and the proposed regimen includes at least 1 drug from each group: 1 from the fluoroquinolone class, also called group 2 (ofloxacin, levofloxacin, or moxifloxacin); 1 from the injectable agents or group 3 (amikacin, kanamycin, or capreomycin); and 2 from group 4 (ethionamide/prothionamide, cycloserine/terizidone, or para-aminosalicylic acid). Of the group 4 drugs, the order of preference is ethionamide, followed by cycloserine and para-aminosalicylic acid. These drugs are added until 4 effective drugs are established.^2^ If 4 drugs are not established or the efficacy of the combination is doubtful, 2 group 5 drugs (clofazimine, amoxicillin clavulanate, linezolid, imipenem, clarithromycin, high-dose isoniazid, or thiacetazone) are selected – 2 of these group 5 drugs are counted as a single effective drug to strengthen the regimen.^3^ The recommended treatment duration is at least 20 months, with a minimum duration of 8 months for the injectable agent.^2^

## MULTIDRUG-RESISTANT TUBERCULOSIS CLINICAL RESEARCH STUDIES IN TANZANIA

We have conducted several MDR-TB studies in Tanzania, aimed at improving treatment through an individualized approach.^4–8^ One of the studies sought to describe the application of second-line drug susceptibility testing (DST) using minimum inhibitory concentration (MIC) results and compare those results with the empirical regimen used during a patient's treatment with a second-line regimen.^5^ MIC allows categorization of isolates near the resistance breakpoint, ‘borderline susceptible’, that may be labelled as ‘susceptible’ by conventional testing but are subject to clinical resistance with poor drug absorption, altered metabolism, or inhibited protein binding. In this study, we found the majority of patients had at least 1 medication that could have been modified with the application of MIC guidance. The most common medication modification in the MDR-TB regimen was changing ethionamide to para-aminosalicylic acid, which could have happened for more than 50% of patients.^5^ Although ethionamide is considered tuberculocidal at higher concentrations, a different study showed that only 22% of subjects had pharmacodynamic indices (serum concentration 2 hours post medication [C_2hr_]/MIC) ratios more than 2.^6^ While ethionamide may have a less predictable time to peak concentration than the other oral agents tested–which may result in an underestimation of the measured C_2hr_ in some subjects–alternative second-line agents, such as para-aminosalicylic acid, may provide a more reliable benefit in the subset of patients with reduced drug exposure and borderline MICs. Additionally, research has shown that 22% of MTB isolates on kanamycin had borderline or resistant MICs, while amikacin retained full susceptibility and, therefore, required substitution within the aminoglycoside class.^5^ WHO recommends to substitute aminoglycosides in case of resistance into polypeptides, such as capreomycin, in group 3.^3^

Fortunately, in all of the studies we have conducted, resistance to the fluoroquinolone class was low, ranging from 5% to 15%; however, 45% of patients had isolates of borderline susceptibility.^4,5,8^ Moreover, 52% of our MDRTB patients had circulating levofloxacin serum drug levels below the expected range when the levofloxacin was given as a 750 mg daily dose.^6^ Recent studies of levofloxacin demonstrate that the best pharmacokinetic properties may be achieved at a dose of 1000 mg daily.^9^ Such optimization may be applicable in our setting, given that no subject in this study was on doses of levofloxacin as high as 1000 mg. The C_2hr_ of levofloxacin for all subjects was below the median maximum serum concentration (C_max_) of 15.5 lg/mL.^6^ Correlating with what we observed, that a high proportion of MDR-TB isolates had borderline susceptibility on ofloxacin in MIC plates, these findings suggest a value to conducting clinical trials to evaluate levofloxacin at a dose of 1000 mg.

We also looked further into the resistance patterns of MDR-TB isolates using genotypic methods in the following mutations: rifampicin (*rpoB*), isoniazid (*inhA or katG*), ethambutol (*embB*), pyrazinamide (*pncA*), ofloxacin (*gyrA*), amikacin (*rrs or eis*), and ethionamide (*inhA*).^5^ The results were compared with MICs, which showed a good correlation to the drugs tested. The few discrepancies showed resistance with MICs but were genotypically wild on the known regions conferring resistance. Although pyrazinamide MIC assays were not conducted, the *pncA* mutation was common, meaning that the suspected pyrazinamide resistance could have impact on treatment outcome.^10^

Our results suggest that in Tanzania, *inhA* and/or *katG* genotypic results could be used to optimize MDR-TB treatment. This can be done by screening MDR-TB with a GenoType MTBDRplus assay (Hain Lifescience GmbH, Nehren, Germany), which identifies *inhA* and/or *katG*. The research suggests that a mutation in the inhA region alone can exclude ethionamide from the MDR-TB empirical regimen, while adding high-dose isoniazid and paraaminosalicylic acid to the regimen. While an exclusive mutation on katG includes ethionamide, a mutation of both *inhA* and *katG* excludes ethionamide and adds para-aminosalicylic acid only. Further research is required to determine the empiric choice of ethionamide or para-aminosalicylic acid while processing for MIC testing.

Given the low proportion of isolates with resistance to injectable agents, the lack of *rrs* or *eis* mutations noted in the isolates, and the borderline susceptibility to ofloxacin, the new GenoType MTBDRsl assay (Hain Lifescience GmbH, Nehren, Germany) for these targets may be of less value. Instead, MIC testing would allow for selection within the class of aminoglycosides and support the use of high-dose levofloxacin.

We, therefore, believe that quantitative susceptibility methods would prove as useful and cost-effective as MDRTB programmes that individualize management based on second-line drug susceptibility. This approach can be made even more cost effective by developing in-house laboratory platforms with the capacity to perform TB cultures.

Our findings on quantitative MICs in MDR-TB patients led to research work on MDR-TB drug concentrations relative to MICs, particularly when compared to the TB Drug Activity (TDA) assay.^6,7^ The TDA assay uses a patient's plasma or serum collected during TB treatment and the patient's own MTB isolate and measures time to detection in liquid culture. Following extensive in-vitro studies, the TDA assay revealed that it predominantly measures the concentration-dependent activity of the aminoglycoside and fluoroquinolone components of the standard MDR-TB regimen.^6^ The study further demonstrated the inactivity of pyrazinamide at the pH of the media used and the comparatively low concentration/MIC achievable for ethionamide and cycloserine. As such, in a patient on a standard MDR-TB regimen in Tanzania, a TDA value approaching 1.0 may be considered to have little plasma-killing potential. If this phenomenon occurs in the aminoglycoside or fluoroquinolone, it is similar to having XDR-TB, which indicates higher mortality and overall treatment failure. While the TDA assay cannot assess drug activity at the site of infection, subjects from Tanzania who had a faster time-to-sputum culture conversion were more likely to have high TDA values.^6,7^ However, it is important to note that plasma drug activity was not exclusively predictive of sputum culture conversion and the TDA assay could not discriminate the relative contribution of individual drugs in the MDR-TB regimen, particularly at the highest TDA values. This may be a consequence of the range of C_2hr_ observed in subjects on the MDR-TB regimen and the unknown target concentration/MIC for a drug such as cycloserine that is not entirely concentration dependent or tuberculocidal in action.

Several limitations were recognized in these studies: critical concentrations for second-line medication still remain a subject of debate and not all regions of mutations or MTB mechanisms for conferring drug resistance have been identified. Despite these limitations, we believe we have established options and evidence for optimization of MDR-TB management in Tanzania. With the scale up of rapid molecular diagnostics – specifically, GeneXpert and GenoType MTBDRplus – in the country, we expect more MDR-TB cases will be diagnosed and effectively treated than have been in the past. Those who need special attention, for instance those harbouring extensively drug-resistant TB (as diagnosed by second-line MIC, or the functional equivalent by TDA with TDA ≤1.0), drug reactions, or delayed culture conversion will continue to be treated at the National Centre of Excellence for MDR-TB management. Previously, we compared the overnight-pooled method to the current standard spot technique for quality, quantity, and time to MTB detection by culture using the BACTEC mycobacterial growth indicator tubes (MGIT) system (BD Diagnostics, Sparks, MD, USA) for pulmonary TB-suspected patients.^11^ The study found that modifications of the overnight-pooled sputum collection method improved the time to detection in the MGIT system among culture-positive samples. This is important not only for identifying susceptibility in phenotypic methods, but also for microbio-logical monitoring of patients during the course of MDR-TB treatment. We, thus, recommend improving sputum collection for efficient monitoring of microbiological responses by use of the overnight-pooled method.

## CONCLUSION AND RECOMMENDATIONS

Examination of second-line DST conducted with Tanzanian MDR-TB patients showed resistance to the important MDRTB drugs, such as ethionamide, ofloxacin, amikacin, kanamycin, and pyrazinamide. Likewise, pharmacokinetic studies on existing MDR-TB regimens showed a high frequency of patients with circulating drug levels below the expected ranges, especially for levofloxacin and kanamycin – 2 key drugs in MDR-TB treatment that also affect ex-vivo plasma drug activity.

To optimize MDR-TB management in Tanzania and similar settings at all levels, we propose the following: patients diagnosed with MDR-TB by either the GenoType MTB/RIF or GenoType MTBDRplus rapid molecular diagnostic test shall submit pretreatment overnight-pooled sputum for culture to establish baseline mycobacteriology for culture-based DST. Next, the empirical second-line (MDR-TB) regimen will be started, but the regimen will be based on *inhA* or *katG* results to determine if ethionamide, para-aminosalicylic acid, or high-dose isoniazid should be added to the empirical regimen. Then, quantitative MIC results for second-line DST should be determined within 2 months of initiation of treatment and used to alter the regimen, if needed. Monthly overnight-pooled sputum should be collected to monitor the time-to-culture conversion. Blood should be taken at weeks 2 to 4 for the TDA assay or alternate therapeutic drug-monitoring (TDM) test. In combination with other standard clinical factors, quantitative second-line drug MIC and TDA/TDM results should be used to tailor the appropriate regimen.
